# Predicting stress response trajectories: Differential contributions of limbic and prefrontal regions to cortisol and affective responses

**DOI:** 10.1038/s41398-026-04140-0

**Published:** 2026-06-12

**Authors:** Renée Lipka, Ludwig Kreuzpointner, Christoph Bärtl, Marina Giglberger, Julian Konzok, Hannah L. Peter, Nina Speicher, Lea Waller, Brigitte M. Kudielka, Stefan Wüst, Henrik Walter, Gina-Isabelle Henze

**Affiliations:** 1 Department of Psychiatry and Neurosciences CCM , Charité – Universitätsmedizin Berlin, Corporate Member of Freie Universität Berlin and Humboldt-Universität zu Berlin, Berlin, Germany; 2 Department of Psychiatry and Neuroscience CBF, Charité – Universitätsmedizin Berlin, Corporate Member of Freie Universität Berlin and Humboldt-Universität zu Berlin, Berlin, Germany; 3 Berlin School of Mind and Brain, Humboldt Universität zu Berlin, Berlin, Germany; 4 German Center for Mental Health (DZPG), Partner Site Berlin - Potsdam, Berlin, Germany; 5 Institute of Psychology, University of Regensburg, Regensburg, Germany

**Keywords:** Molecular neuroscience, Predictive markers, Human behaviour

## Abstract

Why do individuals respond differently to stress? Since rodent studies indicated that stress regulation relies on limbic and medial prefrontal cortex (mPFC) outputs, we aimed to investigate whether data from these regions could also predict cortisol and affect trajectories following psychosocial stress in humans. In this pre-registered study, 281 healthy adults (145 female) were exposed to Scan*STRESS*. Repeated assessments of salivary cortisol and negative affect were used to identify response trajectories (i.e. groups of participants) using latent class mixture modelling (LCMM). LCMMs without brain predictors were compared to LCMMs including structural (volume, thickness) and functional (activation, exposure-time effect) predictors from the amygdala, hippocampus, or mPFC regions, using common fit indices including the Akaike Information Criterion. Results showed that cortisol LCMMs without brain predictors exhibited a single mean trajectory, indicative of homogeneous cortisol responses across the sample. Adding brain predictors resulted in three to four response trajectories, depending on region and outcome. Within identified models, cortisol ‘hyper-response’ trajectories were predicted by larger amygdala and hippocampus volumes. Cortisol ‘non-responses’ were predicted by greater amygdala activation and volume. ‘Elevated baseline’ cortisol was predicted by higher hippocampal activation. mPFC markers did not predict cortisol trajectories, however, medial orbitofrontal cortex parameters identified negative affect response profiles mirroring measures of long-term stress exposure and affect. Together, our findings suggest dissociated roles of limbic and mPFC regions in stress regulation: While limbic structures predicted cortisol responses, the mPFC shaped affective experience.

## Introduction

People respond differently to stress. This variability is evident in both subjective emotional experience as well as cortisol output, the end-product of the hypothalamic-pituitary-adrenal (HPA)-axis. Following exposure to a stressor, the HPA-axis becomes activated with the hypothalamus secreting corticotropin-releasing hormone (CRH), which in turn stimulates adrenocorticotropic hormone (ACTH) release from the pituitary, finally resulting in cortisol secretion from the adrenal glands. The temporal dynamics of this response determine their effect: During the first 20 minutes, cortisol primarily binds to high-affinity mineralocorticoid receptors (MRs), which facilitates risk assessment, memory retrieval, and response selection. Subsequently, rising cortisol levels also engage lower-affinity glucocorticoid receptors (GRs), which promotes memory consolidation and negative feedback terminating HPA axis activity [1, 2]. When individuals cope successfully, this rapid rise and recovery of cortisol promotes neuroplasticity, reinforcing effective coping strategies, thereby forming internal schemes of control and safety, which fosters resilience [2]. In contrast, deviations from the normative response, such as exaggerated, prolonged, or flattened cortisol responses have been associated with a range of stress-related physical and mental health outcomes, including depression, anxiety, chronic pain, and fatigue [3, 4]. Insights into cortisol variability therefore have implications for both prevention and treatment interventions.

Traditionally, variability in cortisol stress responsivity has been investigated by grouping participants into responders and non-responders, or through summary metrics, such as difference scores or areas under the curve [5, 6]. Though useful for data reduction, these methods often mask temporal dynamics or treat potentially meaningful variability as noise. In contrast, latent class mixture modelling (LCMM) aims to directly model this heterogeneity by identifying groups of participants with similar response trajectories, without making a priori assumptions about the size or pattern of such trajectories [7]. In healthy females, LCMM identified three cortisol response types: mild, moderate, and heightened. Interestingly, both mild- and heightened responders reported greater negative affect than moderate responders [8]. This addressed a long-standing challenge in the field, where traditional methods had faced difficulty in consistently linking cortisol and affective responses [9–11]. In males, four cortisol profiles were identified—mild, moderately low, moderately high, and hyper-responses—but these did not differ in negative affect [12]. In females with and without childhood adversity and depression, LCMM identified two profiles: responders and non-responders with high baseline cortisol. Additional analyses showed that only responders successfully adjusted their behavior to changing reward contingencies in a probabilistic task [13]. These findings illustrate that LCMM captures interindividual variability that may go beyond traditional metrics and can reveal links to affect and behavior.

However, understanding of the processes that give rise to such variability is limited. A comprehensive review of rodent literature indicated that although cortisol is secreted peripherally, the magnitude and duration of release is regulated centrally by brain regions including the amygdala, hippocampus, and medial prefrontal cortex (mPFC) [14]. These regions are rich in MRs and GRs and participate in both the initiation and termination of the HPA axis through activation and inhibition of hypothalamic CRH neurons. Chronic stress disrupts this regulation, resulting in hyperactivation of the amygdala and impaired feedback in the hippocampus and mPFC, which was linked to exaggerated- and prolonged cortisol responses, respectively [14]. Given the evolutionary conservation of the HPA system, these mechanisms likely apply to humans as well [14]. However, most human studies have focused on aggregated cortisol metrics, rather than modeling trajectories over time.

Human studies linking brain and cortisol responses have mainly used structural and functional magnetic resonance imaging (sMRI, fMRI). sMRI research investigates gray matter properties of brain regions, with subcortical volumes derived directly from anatomical segmentations and cortical thickness being estimated via surface reconstructions to account for cortical folding [15]. These structural measures have been associated with cortisol stress responses: smaller hippocampal volumes were related to both heightened and flattened cortisol stress responses [8, 16]. Amygdala volume correlated positively with cortisol stress responses in depressed adolescents, but negatively in healthy controls [17]. Although cortical thickness has been studied less frequently in relation to cortisol, reductions in mPFC thickness—especially medial orbitofrontal cortices (mOFC) and rostral anterior cingulate cortex (rACC)—are among the most robust findings in stress-related disorders [18, 19].

Beyond structural measures, fMRI studies have investigated neural activation under stress and control conditions. A prominent example is the Scan*STRESS* paradigm, which aims to elicit psychosocial stress by having participants perform mental rotation- and arithmetic tasks while being submitted to time pressure and social evaluation [20, 21]. A systematic review of such paradigms found that greater mPFC and ACC activation was associated with higher cortisol responses, while findings for the amygdala and hippocampus varied in direction [22]. Moreover, some studies also reported time-dependent activation changes in these regions during stress, known as exposure-time effects, which may reflect sensitization or habituation processes relevant to stress regulation [20, 23–25]. Collectively, these findings further implicate the mPFC, hippocampus, and amygdala in the regulation of cortisol stress responses.

In summary, cortisol stress responses vary across individuals. While a rapid rise and recovery of cortisol levels is generally considered adaptive, deviations from this pattern may confer a latent vulnerability to disease development. LCMM approaches have captured meaningful subtypes of cortisol trajectories, which link to affect and behavior. However, the neurobiological mechanisms underlying these trajectories remain insufficiently understood. While rodent studies suggest a central HPA axis regulation via MR/GR-rich brain regions, most human studies lack the temporal specificity needed to link structure and function with cortisol dynamics. In the present work, we therefore investigated whether structural and functional parameters of key brain regions predict cortisol stress response trajectories identified through LCMM. We focused on four regions of interest (ROIs): the amygdala and hippocampus, based on their established relevance across species [14] and imaging modalities [8, 17, 22], and two mPFC regions—the rACC and mOFC—which have been consistently implicated in structural [18, 19] and functional approaches [22].

Following our pre-registration, for each ROI, we extracted subcortical volume or cortical thickness, task-based activation during Scan*STRESS*, and activation deltas reflecting exposure-time effects. We then used these parameters to predict cortisol trajectories defined by LCMM. We hypothesized that the inclusion of brain parameters may improve identification and prediction of cortisol trajectories beyond what is achieved in LCMMs without such parameters. In pre-registered exploratory analyses, we also investigated whether ROI-informed LCMMs identified negative affect trajectories, and whether these would overlap with cortisol trajectories. We hypothesized that the interposition of brain parameters may help resolve the cortisol-affect incongruency observed using traditional methods. Finally, we explored psychometric properties of the resulting trajectory groupings, as well as assignment coherence across models. We also ran specificity and sensitivity models using brain regions from the visual cortex or including total brain volume (TBV) as an additional predictor. These final analyses were not pre-registered but added to explore the functional meaning, reliability, specificity, and sensitivity of the identified trajectories.

## Materials and methods

### Design

**Fig. 1:**
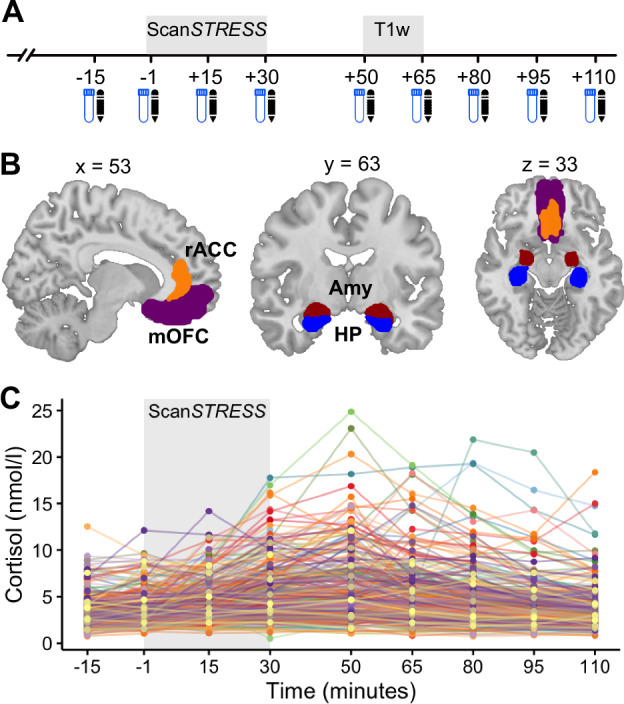
Experimental procedure, regions of interest (ROIs), and baseline variability prior to cortisol trajectory estimation. **A** Experimental procedure consistent across studies. Blue icons represent salivary cortisol samples. Black pens represent negative affect samples. **B** ROIs from which three parameters were extracted per participant (volume/thickness, activation, activation delta i.e., exposure-time effect). **C** Variability in cortisol responses across the sample. Abbreviations: Amy, amygdala (red); HP, hippocampus (blue); mOFC, medial orbitofrontal cortex (purple); rACC, rostral anterior cingulate cortex (orange).

The study was pre-registered (https://osf.io/gzh2j/) and conduced a secondary analysis of five studies investigating healthy participants with Scan*STRESS* (*N* = 281; 145 females, mean age 25.21 ± 7.65). Exclusion criteria included any current or past psychiatric-, neurological-, or endocrine disorder; use of medication (except hormonal contraception) or other substances affecting the central nervous system; tobacco consumption (>5 cigarettes/day); shift work; and MRI contraindicators. Test sessions took place between lunchtime and afternoon (11.30–6 pm) and included a 45-min relaxation period to circumvent diurnal and activity-related effects on cortisol. All studies were approved by the local ethics committee of Regensburg university and participants gave written informed consent [20, 23, 26–28]. Scan*STRESS* was presented as a block-design with alternating stress and control blocks. After the first half of the task (run1), participants received intercom feedback that their performance was below average, urging them to extend more effort to salvage data of the second half (run2) (see [20, 21] for details). Cortisol and affect were sampled across nine timepoints surrounding stressor onset (*t* = −15, −1, +15, +30, +50, +65, +80, +95, +110 mins), and T1-weighted structural images were acquired following the task (Fig. 1A). Salivary cortisol samples were taken using salivette cotton swaps (Sarstedt, Nuembrecht, Germany). Affect was captured using the Positive and Negative Affect Schedule (PANAS), though only the negative affect dimension was used for analyses, as it is particularly sensitive to aversive mood states and is strongly associated with subjective distress [29]. We refer to the original publications for further details on data collection and our pre-registration for details on protocols and data selection.

### (f)MRI preprocessing and analyses

All MRI data were acquired at Regensburg University, Germany, using a 3T Siemens MAGNETOM Prisma scanner with 64-channel head coil (Siemens Healthcare, Erlangen, Germany). Preprocessing and statistical analyses were performed using *HALFpipe* [30, 31]. Structural images were skull stripped using the convolutional neural network *SynthSeg* [32]. Preprocessing of functional data involved motion correction (volume resampling), bias field correction, co-registration, registration to standard space, spatial smoothing (6 mm), grand mean scaling, and high pass filtering at 125 s. For statistical analyses, atlas-based parameters of structure, activation, and exposure-time effects were extracted in a lateralized fashion for the four ROIs: amygdala, hippocampus, rACC, mOFC using Desikan-Killiany and Destrieux atlases [33, 34]. Structural parameters were derived from T1-weighted images via *FreeSurfer* surface- and volume-based processing streams (i.e. volume for subcortical regions and thickness for cortical regions) [35, 36]. Functional parameters were derived from intraindividual contrasts within general linear models (*stress* > *control*). This was done in two ways: (1) globally over both runs of Scan*STRESS*, yielding mean activation, and (2) separately for each run, calculating the difference (*run*2 − *run*1), yielding activation deltas representing exposure-time effects. We note that we used motion regression rather than the pre-registered *ICA-AROMA* for model correction, as it performs less aggressive cleaning and potential data distortion [37]. In summary, for each participant we derived three parameters per ROI: a structural parameter (volume (mm^3^)/thickness (mm)), mean activation (*z*-score), and exposure-time effect (*z*-score-delta). See Fig. 1B for a visualization of ROIs.

### LCMM analyses

Figure 1C displays baseline variability in cortisol responses across the sample. To identify latent trajectories (i.e., groups of participants) exhibiting similar responses, we fit latent process mixed models using the R package *lcmm* (v 2.1.0) [7, 38]. Model estimation was done using a grid of initial values to ensure convergence toward the global maximum (100 departures, 30 iterations) [7]. First, we fit baseline models with 1–4 latent trajectories, including *age* and *sex* as covariates. This way, only residual heterogeneity in cortisol was explored after accounting for known factors of change over time [39, 40]. Note that we controlled for *sex* and not hormonal status since the latter had no influence on cortisol responses in our sample (see supplementary section ‘hormonal status’).

In a second step, we explored whether adding brain parameters would improve model fit and predict cortisol trajectory assignment. To this end, 2–4 trajectory models were fit for each ROI, adding structure, activation, and exposure-time effects as predictors of trajectory membership, while still correcting for *age* and *sex*. To account for differences in scale, predictors were z-standardized prior to model estimation. Trajectory predictors entered a polynomial logistic regression model, predicting trajectory membership from brain parameters. This included the choice of a reference trajectory, against which the odds of belonging to each of the other trajectories were estimated [41].

For model comparison, we used common fit indices: Bayesian Information Criterion (BIC), size adjusted BIC (SABIC), and Akaike Information Criterion (AIC), which each indicate better fit with lower values. AIC and SABIC involve smaller penalties for model complexity. In samples <400 individuals, BIC tends to penalize strongly for complexity in relation to sample size and is therefore biased towards selecting fewer trajectory models [42]. Hence, the AIC is assumed to be the better selection criterion for our sample size [43]. Entropy was consulted as a measure of discriminatory power, ranging between zero and one, with higher values indicating greater certainty in trajectory assignment across participants [43]. There are no agreed upon cutoffs for entropy values [44]. Values close to one are considered optimal in some cases [44] but may indicate overfitting in others [43]. Entropy values should therefore only be used as orientation and not to determine the best model [43]. Nevertheless, a common heuristic is to consider entropies >0.8 acceptable [44]. Finally, models were also examined for their compatibility with current theory [1–3].

To verify that trajectories of selected models were distinguishable, we ran repeated-measures analyses of variance (ANOVAs) with *time* as within- and *trajectory* as between-subjects factors. Though this approach may seem circular, it is considered the most appropriate post-hoc test for LCMMs [8, 45]. Note that we will refer to trajectories as ‘hyper’ and ‘hypo’ responses to reflect relative sample differences and maintain consistent terminology across studies [8, 12], but do not intend to imply clinical (mal)adaptation.

In non-pre-registered analyses, we further explored the functional meaning of identified trajectories using chi-square tests and repeated-measures ANOVAs on *age*, *sex*, long-term affect using the Beck Depression Inventory (*BDI-II*) [46] and Anxiety Sensitivity Index (*ASI*) [47], as well as indices of chronic stress using the screening scale of the Trier Inventory for the assessment of Chronic Stress (*TICS*) [48], Life Events Checklist (*LEC*) [49], and Childhood Trauma Questionnaire (*CTQ*) [50]. Using Cohen’s and Fleiss’ kappa, we further explored trajectory assignment coherence across different models (i.e. between ROIs and cortisol/affect).

To test the regional specificity of our findings, we also repeated brain models using visual cortical regions as predictors of trajectory membership. More specifically, we ran two additional model sets using data from calcarine- and cuneal cortices, which are not commonly associated with stress regulation [14, 25]. These analyses were not pre-registered and described in more detail in supplementary section ‘visual cortex regional specificity analyses'.

Due to convergence issues, we deviated from our pre-registration in two additional points. First, as brain size influences regional volumes, especially of subcortical structures [51] we aimed to include TBV as an additional covariate in subcortical models. However, this would have corrected cortisol trajectories, rather than brain parameters. Instead, we re-ran subcortical models with TBV as an additional trajectory-membership predictor, adjusting the effect of other predictors for total brain size. Due to the added complexity, these models faced convergence difficulties, and converged solutions exhibited reduced parameter precision and increased uncertainty of some estimates (see supplementary section ‘TBV subcortical sensitivity analyses’).

Secondly, we had planned to estimate separate models for left and right ROIs. However, this complicated model comparison, as lower-entropy solutions showed limited assignment coherence across sides, despite visually comparable trajectories. To circumvent this, we first included a side variable as interaction terms of brain parameters, which increased complexity and non-convergence. Finally, given high correlations between left and right parameters (supplementary Fig. S1), we merged sides per ROI, aiding convergence and model selection. Following model estimation, we nevertheless plotted left and right parameters individually, yielding similar patterns across sides (see Figs. 3, 4, and 6C–E).

### Exploratory affect analyses

As pre-registered exploratory affect analyses, we first entered cortisol trajectory groupings from selected brain models into repeated-measures ANOVAs on the PANAS negative affect dimension, with *time* as within- and *trajectory* as between-subjects factors. When cortisol trajectory groupings did not explain variance in negative affect, we tested whether re-running LCMMs on negative affect would identify affect trajectories that corresponded meaningfully with cortisol trajectories. Therefore, we replicated baseline and brain models described in the ‘LCMM analyses’ section using negative affect as the outcome.

**Fig. 2:**
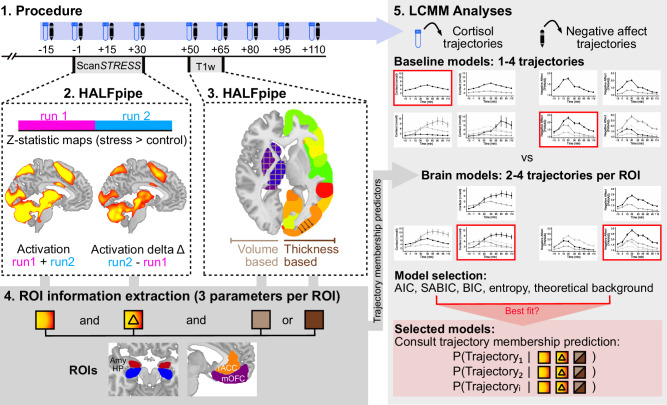
Roadmap of the analytic pipeline. **1** Each participant underwent Scan*STRESS* and T1-weighted structural imaging, while giving multiple salivary cortisol (blue icons) and negative affect samples (black pens). **2** Functional and structural data were preprocessed and analyzed using *HALFpipe*. Following preprocessing (not shown), functional data were analyzed using general linear models (*stress* > *control*) and creating individual *z*-statistic maps by contrasting run1 and run2 in different ways: *run*1 + *run*2 = *mean activation*, *run*2 − *run*1 = *activation delta*. **3** Structural data was parcellated into anatomical regions to extract subcortical volumes (beige) and cortical thickness (brown). For subcortical regions, local gray matter volume can be extracted by summing over gray matter voxels (3D pixels) within each anatomical label. In contrast, cortical analyses must account for its sheet-like geometry by estimating distance between the inner (white matter) and outer (cerebrospinal fluid) surfaces (i.e. cortical thickness). **4** Following *HALFpipe* analyses, we selected information from four regions of interest (ROIs): the amygdala (Amy), hippocamps (HP), rostral anterior cingulate (rACC) and the medial orbitofrontal cortex (mOFC). Specifically, for each ROI we extracted two functional parameters: mean activation (yellow-red box) and activation delta (yellow-red Δ-box), as well as one structural parameter: either subcortical volume (beige box) or cortical thickness (brown box), depending on whether the ROI was at a cortical- or subcortical location. The three ROI-specific parameters were then entered into subsequent latent class mixture models (LCMMs). **5** LCMMs identify latent trajectories in longitudinal data. We took cortisol and negative affect samples acquired during the scanning procedure to identify latent cortisol and negative affect trajectories. This was done in two broad steps: First, we ran 1–4 trajectory baseline models, without additional brain parameters, only correcting for *age* and *sex*. Then, we ran 2–4 trajectory brain models including the three brain parameters, separately for each ROI. Models were selected based on common fit indices (AIC, SABIC, BIC, entropy) and an examination of their theoretical plausibility. Baseline models were contrasted with brain models from each ROI. For cortisol models, we display the results of baseline and amygdala models as one example. Among baseline models, the 1-trajectory model had the best fit, which was however outperformed by the 4-trajectory solution when adding amygdala parameters. For negative affect models, the best-fitting baseline model yielded three trajectories, which was outperformed by the 4-trajectory model with parameters from the mOFC ROI, as displayed. Following model selection of brain models, we consulted the polynomial logistic regression part of these models, which aims to predict trajectory membership using the three brain parameters as predictors. Specifically, it calculates the probability of belonging to each of the trajectories (i) given the set of brain parameters.

Together, LCMM comprises the estimation of multiple models before selecting best fitting ones. We estimated 16 cortisol models and 16 negative affect models, which were pre-registered, and out of which we selected those which outperformed the baseline model (two cortisol models and one negative affect model). See Fig. 2 for a visualization of the pre-registered analytic pipeline. We further ran six regional specificity and six limbic TBV sensitivity models, which were not pre-registered, and out of which we explored four (one for each region). In total, we estimated 44 models and selected seven for further analyses.

## Results

### Baseline models

When comparing the four baseline cortisol models (corrected for *sex* and *age*), all model comparison indices (AIC, BIC, SABIC, entropy) favored the single-trajectory solution (Table 1). In the following, we compare models with brain parameters to this best-performing baseline model.

**Table 1:** Between model comparison of cortisol models.

Model	Loglik^a^	Npm^b^	AIC	BIC	SABIC	Entropy	Trajectory size (%)			
							1	2	3	4
**Baseline models**										
1-Trajectory	−4629.33	14	**9286.67**	**9337.61**	**9293.22**	**1.00**	100	NA	NA	NA
2-Trajectory	−4628.63	16	9289.27	9347.48	9296.75	0.92	99.28	0.71	NA	NA
3-Trajectory	−4625.61	19	9289.23	9358.36	9298.11	0.66	81.85	17.08	1.06	NA
4-Trajectory	−4622.34	22	9288.68	9368.72	9298.96	0.60	41.63	10.67	39.14	8.54
**Amygdala models**										
2-Trajectory	−4625.05	19	9288.10	9357.23	9296.99	0.85	4.27	95.72	NA	NA
3-Trajectory	−4618.76	25	9287.52	9378.47	9299.20	0.56	61.92	6.04	32.02	NA
4-Trajectory	−4603.81	31	**9269.63**	9382.42	**9284.12**	0.71	6.40	4.27	23.13	66.19
**Hippocampus models**										
2-Trajectory	−4625.09	19	9288.19	9357.32	9297.07	0.95	3.91	96.08	NA	NA
3-Trajectory	−4616.37	25	**9282.74**	9373.69	9294.42	0.86	3.91	4.27	91.81	NA
4-Trajectory	−4614.13	31	9290.26	9403.04	9304.75	0.66	3.91	78.64	11.74	5.69
**mOFC models**										
2-Trajectory	−4626.75	19	9291.51	9360.63	9300.39	0.56	88.96	11.03	NA	NA
3-Trajectory	−4622.90	25	9295.81	9386.77	9307.49	0.51	72.59	14.94	12.45	NA
4-Trajectory	−4615.65	31	9293.30	9406.09	9307.79	0.73	11.03	22.06	10.32	56.58
**rACC models**										
2-Trajectory	−4627.96	19	9293.93	9363.06	9302.81	0.51	80.07	19.92	NA	NA
3-Trajectory	−4622.57	25	9295.15	9386.11	9306.84	0.68	74.73	1.42	23.84	NA
4-Trajectory	−4620.25	31	9302.51	9415.30	9317.00	0.49	41.99	20.99	9.25	27.75

^a^Log likelihood, ^b^Number of parameters, bold faced parameters indicate best-fitting models: among the baseline models the 1-trajectory solution provided the best fit, adding brain parameters of the amygdala and hippocampus improved model fit (according to the AIC) over the 1-trajectory baseline model in 4- and 3-trajectory solutions, respectively. Adding mOFC and rACC models did not improve model fit over the 1-trajectory baseline model.

### Brain models

**Fig. 3:**
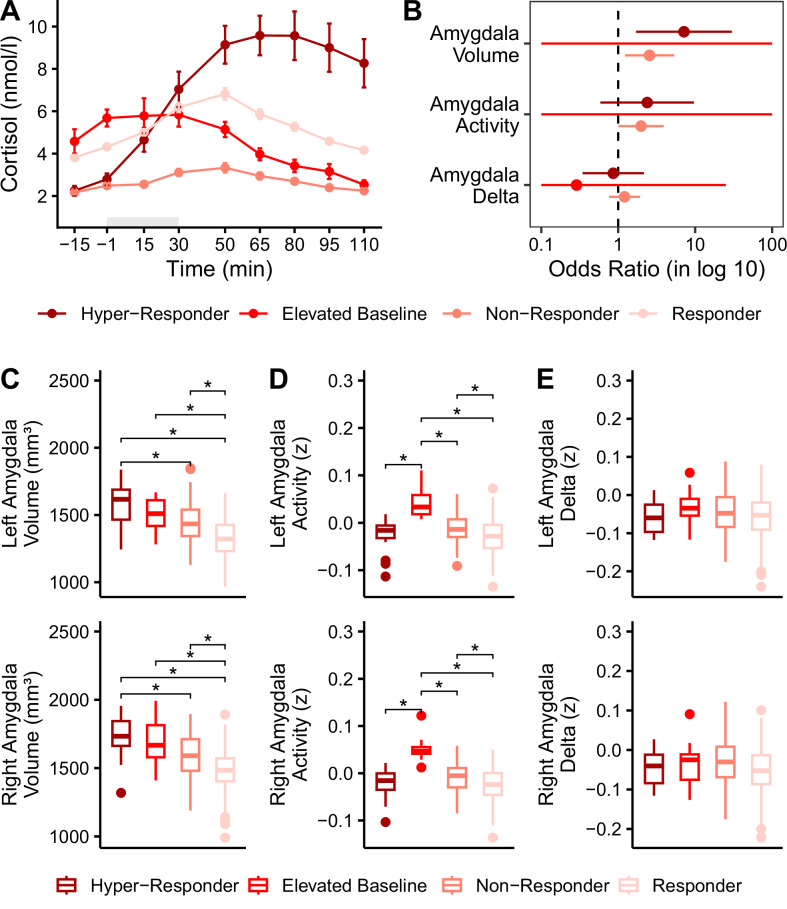
The four-trajectory amygdala model. **A** Mean cortisol across trajectories. Grey shading indicates Scan*STRESS* timing. **B** Amygdala parameters as predictors of trajectory membership. Odds Ratios (ORs) were computed as compared to the reference trajectory (‘responder’). The analysis accounted for the uncertainty (entropy) in trajectory assignment. Note that confidence intervals of the ‘elevated baseline’ trajectory were large and clipped at [0.1, 100] for plotting. **C-E**. Boxplots of amygdala parameters (volume, activation, activation delta (i.e., exposure-time effect), respectively) across trajectories. For each plot, left amygdala parameters are displayed on top, right parameters below. Within plots, statistically significant pairwise comparisons are displayed while non-significant comparisons are left blank.

Among the amygdala models, AIC and SABIC favored the four-trajectory model (*entropy* = 0.72, Table 1). The model aligned well with current theory [3, 4] and showed similarity with existing literature on latent cortisol trajectories [8, 12]. See Fig. 3A for a depiction of the selected model. A repeated-measures ANOVA verified that the cortisol trajectories were distinguishable (main effect of time: $F\left(3.20,\,822.94\right)=26.70,{p} < 0.001,\,{\eta }_{g}^{2}=0.09$, main effect of trajectory: $F\left(3.00,\,257.00\right)=37.85,{p} < 0.001,{\eta }_{g}^{2}=0.31$, time*trajectory interaction $F\left(9.61,\,822.94\right)=15.26,{p} < 0.001,{\eta }_{g}^{2}=0.15)$. The first trajectory showed a heightened cortisol stress response with slowed recovery (‘hyper-responder’, *N* = 18), while the second trajectory was characterized by higher basal cortisol levels and no stress response (‘elevated baseline’, *N* = 12). The third trajectory exhibited a non-response profile (‘non-responder’, *N* = 65) while the fourth trajectory showed a moderate cortisol stress response, with recovery to baseline two hours later (‘responder’, *N* = 184). For trajectory membership prediction, we selected this ‘responder’ trajectory as the reference, as it represents an adaptive response [4], to which aberrant responses could be compared. Results showed that higher amygdala volume increased the odds of ‘hyper-responses’ (${coef}=1.96,{SE}=0.73,{Wald}=2.68,{p} < 0.001,{OR}[95 \% {OR}{-}{CI}]=7.14[1.70,\,29.97]$ or ‘non-responses’ $({coef}=0.93,{SE}=0.37,{Wald}=2.50,{p}=0.01,{OR}\left[95 \% {OR}{-}{CI}\right]=2.55[1.22,\,5.31])$. Amygdala activation also predicted trajectory membership: Compared to ‘responders’, higher amygdala activation increased the odds of cortisol ‘non-responses’ $({coef}=0.68,{SE}=0.34,{Wald}=1.97,{p}=0.05,{OR}[95 \% {OR}{-}{CI}]=1.97[\mathrm{1.013.89}])$. Exposure-time effects did not predict trajectory membership (supplementary Table S1). See Fig. 3B for a depiction of amygdala parameter odds ratios (ORs) per trajectory and 3C-E and supplementary Table S2 for mean amygdala parameters stratified by side and trajectory). Supplementary Table S3 shows that ‘hyper-responder’ and ‘elevated baseline’ groups were predominantly male, while no group differences were observed on other questionnaires.

**Fig. 4:**
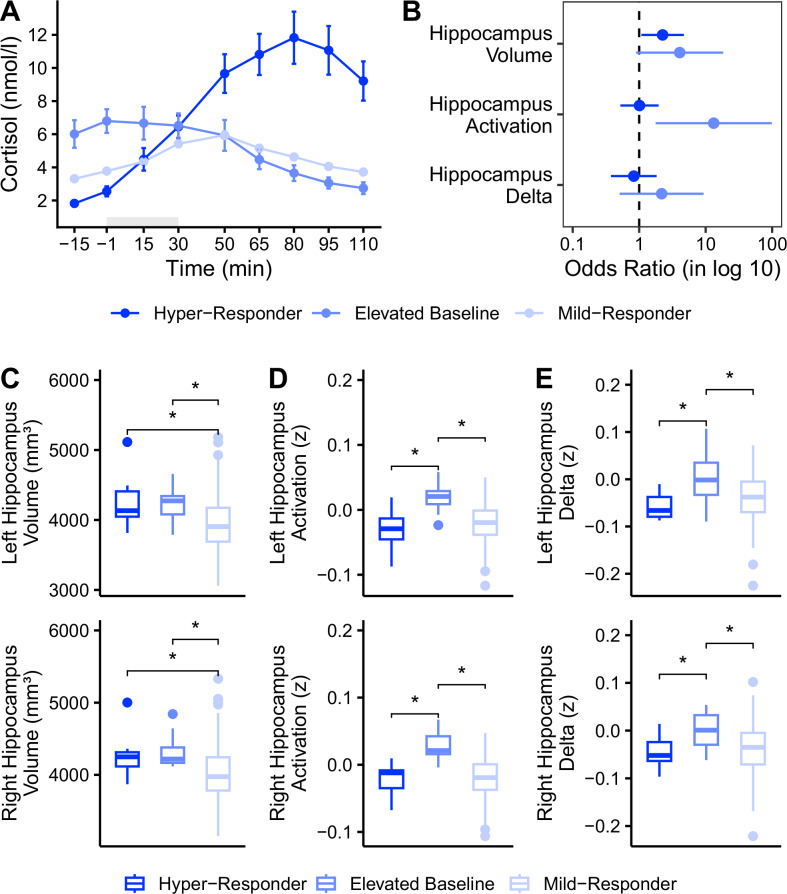
The three-trajectory hippocampus model. **A** Mean cortisol across trajectories. Grey shading indicates Scan*STRESS* timing. **B** Hippocampus parameters as trajectory membership predictors. Odds Ratios (ORs) were computed as compared to the reference trajectory (‘mild responder’). The analysis accounted for the uncertainty (entropy) in trajectory assignment. **C-E** Boxplots of hippocampus parameters (volume, activation, activation delta (i.e., exposure-time effect), respectively) across trajectories. For each plot, left hippocampus parameters are displayed on top, right parameters below. Within plots, statistically significant pairwise comparisons are displayed while non-significant comparisons are left blank.

Concerning the hippocampus models, AIC favored the three-trajectory model with good entropy (0.86; Table 1) and theoretical plausibility [3, 4] (Fig. 4A). A repeated-measures ANOVA again verified that trajectories were distinguishable (main effect of time: $F\left(3.11,\,802.97\right)=22.91,{p} < 0.001,{\eta }_{g}^{2}=0.08$, main effect of trajectory: $F\left(2.00,\,258.00\right)=12.23,{p} < 0.001,{\eta }_{g}^{2}=0.09$, time*trajectory interaction: $F\left(6.23,\,802.97\right)=28.92,{p} < 0.001,{\eta }_{g}^{2}=0.18$). The first trajectory showed a heightened cortisol response with slowed recovery (‘hyper-responder’, *N* = 11) and the second trajectory exhibited elevated baseline cortisol but non-response to the stressor (‘elevated baseline’, *N* = 12). The third trajectory showed a mild cortisol stress response, with rapid return to baseline two hours later (‘mild responder’, *N* = 254). We again chose the ‘mild responder’ trajectory as the reference for polynomial logistic regression. Results showed that higher hippocampus volume was related to higher odds of a cortisol ‘hyper-response’ $({coef}=0.80,{SE}=0.37,{Wald}=2.14,{p}=0.03,{OR}[95 \% {OR}{-}{CI}]=2.24[1.07,\,4.71])$. Additionally, higher hippocampus activation increased the odds of membership in the ‘elevated baseline’ over the ‘mild-responder’ trajectory $({coef}=2.57,{SE}=1.02,{Wald}=2.51,{p}=0.01,{OR}[95 \% {OR}{-}{CI}]=13.18[1.76,\,98.33])$. Exposure-time effects again did not predict trajectory membership (supplementary Table S4). See Fig. 4B for a depiction of hippocampus parameter ORs per trajectory. Figure 4C–E and supplementary Table S5 shows hippocampus parameter means across sides and trajectories. Table 2 shows that ‘hyper-responder’ and ‘elevated baseline’ groups were predominantly male. Additionally, the ‘elevated baseline’ group reported significantly higher *CTQ* scores than other trajectories, though still remaining in the ‘low to moderate’ severity quintile [52]. No group differences were observed on other variables.

**Table 2:** Characterization of hippocampus model derived cortisol classes.

	Trajectory mean (SD)					
	Hyper-responder	Elevated baseline	Mild-responder	Statistic(df)	*p*	Effect size
Age	23.45 (±3.11)	28.50 (±9.01)	25.14 (±7.70)	F(2, 278) = 1.41	0.24	η² = 0.01
Sex				χ²(2) = 16.34	<0.01*	V = 0.24
M	11	9	116			
F	0	3	142			
BDI-II	9.18 (±10.03)	11.25 (±9.62)	13.56 (±11.69)	F(2, 272) = 0.95	0.38	η² = 0.01
TICS	15.64 (±6.28)	13.50 (±7.62)	18.00 (±9.63)	F(2, 276) = 1.57	0.20	η² = 0.01
ASI (Total)	15.00 (±7.54)	19.67 (±11.44)	20.13 (±10.92)	F(2, 273) = 1.19	0.30	η² = 0.01
CTQ (Total)	29.00 (±4.34)	37.00 (±10.55)	32.15 (±7.35)	F(2, 274) = 3.54	0.03*#	η² = 0.03
LEC (Total)	67.36 (±6.02)	70.75 (±8.07)	67.09 (±12.74)	F(2, 278) = 0.50	0.60	η² = 0.00

*ASI* anxiety sensitivity index, *BDI-II* beck depression inventory II, *CTQ* childhood trauma questionnaire, *LEC* life events checklist, *TICS* trier inventory for the assessment of chronic stress (screening scale). *indicate statistically significant differences between groups. # Pairwise comparisons: hyper-responder < elevated baseline (*p* = 0.02), hyper-responder < mild-responder (*p* = 0.04).

**Fig. 5:**
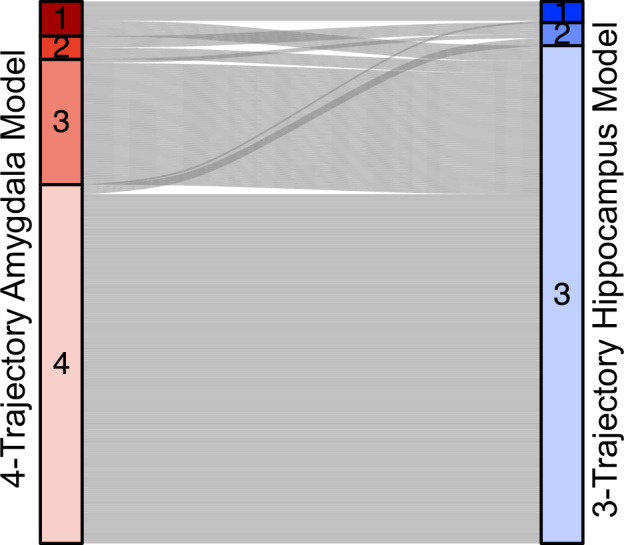
Alluvial plot of participant flow between amygdala (left side) and hippocampus (right side) cortisol models.

In follow-up analyses we assessed the overlap between amygdala and hippocampus models. Visual inspection (Figs. 3A and 4A) suggested that the amygdala ‘responder’ and ‘non-responder’ trajectories had merged into one hippocampus ‘mild responder’ trajectory. To test this, we joined amygdala ‘responders’ and ‘non-responders’ and compared this to hippocampus assignments, yielding moderate agreement (${Cohen}\mbox{'}{s\; Kappa}=0.57,{z}=12.50,{p} < 0.001$). This indicates substantial overlap between trajectories across models, with only a few individuals being reclassified (Fig. 5).

For the mOFC and rACC models, none of the fit indices favored higher trajectory numbers over the single-trajectory solution without additional brain predictors (Table 1). We therefore decided not to explore these models further.

Finally, we also conducted non-preregistered specificity and sensitivity analyses to demonstrate that our cortisol results were specific to limbic regions and remained largely unchanged after adjustment for brain size (see supplementary sections ‘visual cortex regional specificity analyses’ and ‘TBV subcortical sensitivity analyses’ for detailed results).

### Exploratory affect analyses

**Fig. 6:**
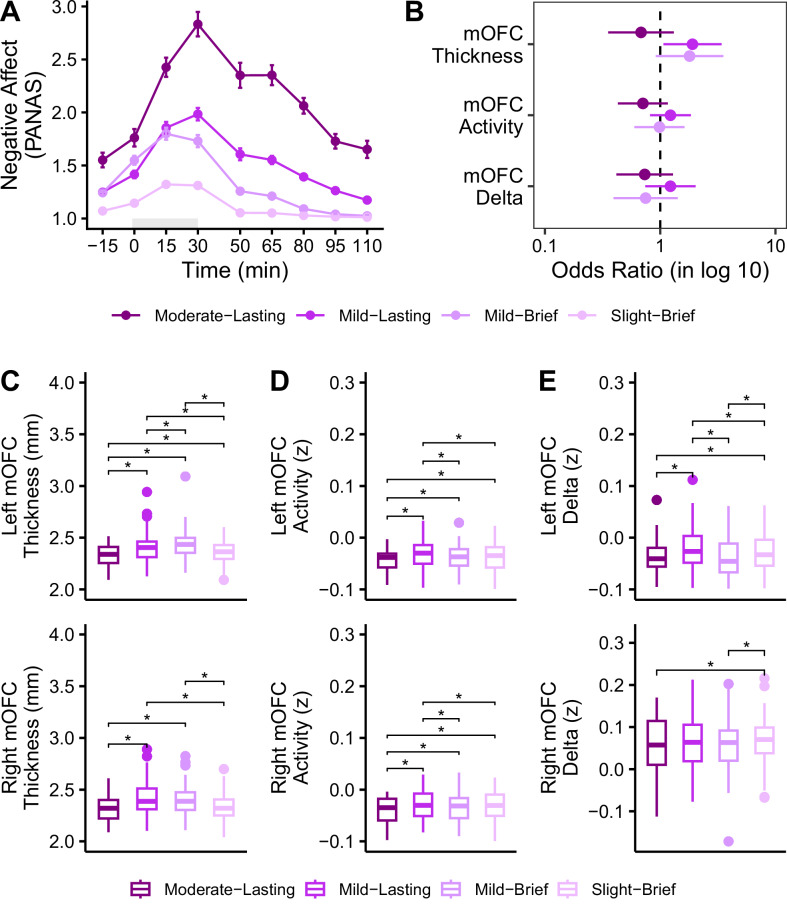
The four-class medial orbitofrontal cortex (mOFC) negative affect model. **A** Mean negative affect across trajectories. Grey shading indicates Scan*STRESS* timing. **B** mOFC parameters (thickness, activation, activation delta (i.e., exposure-time effect)) as trajectory membership predictors. Odds Ratios (ORs) were computed as compared to the reference trajectory (‘mild-brief’). The analysis accounted for the uncertainty (entropy) in trajectory assignment. **C-E** Boxplots of mOFC parameters across trajectories. For each plot, left mOFC parameters are displayed on top, right parameters below. Statistically significant pairwise comparisons are displayed within plots, non-significant ones are left blank.

Next, we explored whether cortisol groups derived from amygdala and hippocampus brain models would explain variance in stress-related negative affect. Repeated-measures ANOVAs indicated that brain-derived cortisol trajectories did not differ in negative affect (see supplementary section ‘exploratory affect analyses’ for statistical detail). We then explored whether running baseline and brain models on negative affect data would identify affect trajectories that corresponded with cortisol trajectories. Results indicated that while distinct negative affect trajectories were identified with and without brain predictors, these did not overlap with cortisol trajectories (supplementary Table S10, Figs. S5, S6). Additionally, amygdala, hippocampus, and rACC predictors showed no utility in predicting negative affect trajectories (all $p\mbox{'}s > 0.05$). However, among mOFC affect models, AIC and SABIC favored the four-trajectory model with *entropy* = 0.67 (supplementary Table S10). The four trajectories followed a similar pattern but varied in negative affect intensity (Fig. 6A). To name trajectories we followed the scaling of PANAS negative affect items, where 1 represents ‘very slightly or not at all’, 2 represents ‘a little’, 3 represents ‘moderately’, 4 represents ‘quite a bit’, and 5 represents ‘extremely’ [29]. Consequently, we termed trajectories ‘slight-brief’ (*N* = 109), ‘mild-brief’ (*N* = 69), ‘mild-lasting’ (*N* = 69), and ‘moderate-lasting’ (*N* = 21) negative affect. For the polynomial logistic regression, we chose the ‘slight-brief’ trajectory as the reference due its resemblance to ‘positive appraisal styles’ associated with resilience [10]. Results showed that compared to this reference, greater mOFC thickness increased the odds of ‘mild-lasting’ trajectory assignment $({coef}=0.64,{SE}=0.29,{Wald}=2.17,{p}=0.02,\,{OR}\left[95 \% {OR}{-}{CI}\right]=1.90\left[1.06,\,3.40\right])$. No other mOFC parameter predicted trajectory membership (supplementary Table S11). See Fig. 6B for mOFC parameter ORs and Fig. 6C–E for descriptive boxplots stratified by side and trajectory. Questionnaire analyses indicated that the intensity of acute stress-related affect was closely mirrored by longer-term measures of stress propensity: while the ‘slight-brief’ trajectory scored lowest on depression, anxiety, life stress, and childhood trauma, more pronounced negative affect trajectories had incrementally higher scores on these scales (Table 3). We do note that this model had an entropy of 0.67, which is indicative of trajectory assignment ambiguity. Though ORs displayed in Fig. 6B are corrected for such ambiguity, we nevertheless urge a cautious interpretation of these results.

**Table 3:** Characterization of mOFC model-derived negative affect trajectories.

	Trajectory mean (SD)						
	Moderate-lasting	Mild-lasting	Mild-brief	Slight-brief	Statistic(df)	*p*	Effect size
Age	23.87 (±3.66)	24.86 (±7.21)	25.62 (±8.19)	25.69 (±8.43)	F(3, 265) = 0.29	0.63	η² = 0.01
Sex					χ²(3) = 3.35	0.34	V = 0.10
M	11	32	38	52			
F	20	37	31	57			
BDI-II	21.71 (±11.33)	17.53 (±12.54)	11.24 (±9.59)	9.36 (±9.91)	F(3, 271) = 15.63	<0.01*	η² = 0.15
TICS	26.32 (±7.43)	21.36 (±9.06)	16.34 (±8.59)	13.81 (±8.45)	F(3, 273) = 22.94	<0.01*	η² = 0.20
ASI (Total)	30.77 (±11.18)	23.03 (±10.76)	19.81 (±9.18)	14.90 (±8.74)	F(3, 270) = 24.59	<0.01*	η² = 0.21
CTQ (Total)	34.58 (±8.05)	33.79 (±7.75)	31.99 (±8.46)	30.70 (±6.10)	F(3, 273) = 3.65	0.01*	η² = 0.04
LEC (Total)	68.00 (±9.14)	66.56 (±10.82)	68.09 (±12.18)	68.20 (±10.99)	F(3, 275) = 0.36	0.78	η² = 0.00

*ASI* anxiety sensitivity index, *BDI-II* beck depression inventory II*, CTQ* childhood trauma questionnaire, *LEC* life events checklist, *TICS* trier inventory for the assessment of chronic stress (screening scale). *Significant pairwise comparisons across questionnaires. **BDI-II:** moderate-lasting > mild-brief, moderate-lasting > slight-brief, mild-lasting > mild-brief, mild-lasting > slight-brief (all *p*’s < 0.01). **TICS:** moderate-lasting > mild-brief (*p* < 0.01), moderate-lasting > slight-brief (*p* < 0.01), moderate-lasting > mild-lasting (*p* = 0.03), mild-lasting > mild-brief (*p* < 0.01), mild-lasting > slight-brief (*p* < 0.01). **ASI:** moderate-lasting > mild-brief, moderate-lasting > slight-brief, moderate-lasting > mild-lasting, mild-brief > slight-brief, mild-lasting > slight-brief (all *p*’s < 0.01). **CTQ:** moderate-lasting > slight-brief (*p* = 0.05), mild-lasting > slight-brief (*p* = 0.03).

## Discussion

Varied cortisol stress responses can be captured using LCMM. Here, we explored whether adding functional and structural brain parameters from the amygdala, hippocampus, rACC, and mOFC would aid the identification of cortisol stress response trajectories. Without brain predictors, the single-trajectory model showed the best fit, indicating largely homogeneous cortisol responses across the sample. The inclusion of amygdala and hippocampus parameters improved model fit and yielded more nuanced solutions (three and four trajectories, respectively), suggesting that interindividual differences in limbic structure and function meaningfully contributed to the identification of divergent stress responses. These effects remained when including TBV as an additional trajectory predictor. In contrast, models incorporating rACC and mOFC parameters did not outperform the baseline cortisol model, nor did parameters from control regions in the visual cortex. However, mOFC parameters aided the identification of negative affect trajectories with similar pattern but varying intensity. We elaborate on these findings in the sections below.

### Amygdala models

The best-fitting amygdala model identified four cortisol response trajectories, which were comparable to previous investigations in number and size [8, 12]. Greater amygdala volume increased the odds of cortisol ‘hyper-responses’ and ‘non-responses’. This was also the case when including TBV as an additional trajectory predictor. Greater amygdala activation also increased the odds of cortisol ‘non-responses’, though this effect was no longer statistically significant in TBV-corrected models.

#### Amygdala volume and 'hyper-responses'

Larger amygdala volumes predicting ‘hyper-responses’ would not be unexpected in a group solely comprised of men. Men often have larger brains, hence larger amygdalae, and generally tend to exhibit greater cortisol stress responses than females [39, 53]. Additionally, participants in this trajectory did not differ in reported life events, psychiatric symptoms, or chronic stress indices. One interpretation of our findings is thus that they reflect healthy men with normative—yet relatively larger—amygdala volumes and cortisol responses. However, our TBV-sensitivity analyses suggest this effect was not driven by sex or total brain size alone. In the literature, cortisol ‘hyper-responses’ are common in depression and anxiety, and thought to result from prolonged HPA-axis activity [3]. The amygdala plays a key role in such activation by disinhibiting hypothalamic CRH-neurons [14]. In rodents, prolonged stress leads to dendritic growth and increased spine density in the amygdala, heightening vigilance and stress responsivity [54]. In healthy humans, greater life stress was also linked to larger amygdala volumes [55, 56] and exaggerated cortisol responses [12]. An alternative read of our results could therefore be that amygdala hypertrophy may drive excess cortisol release, possibly indicating stress system sensitization even before subjective symptoms emerge.

#### Amygdala parameters and 'non-responses'

Interestingly, greater amygdala volume also increased the odds of cortisol ‘non-responses’ and the effect remained when including TBV as an additional trajectory predictor. Cortisol non-responses are assumed to arise secondarily to prolonged cortisol exposure, due to self-preserving compensatory mechanisms resulting in GR feedback hypersensitivity and inefficient stress system mobilization [3]. They are common in posttraumatic stress disorder (PTSD) or following early life adversity, and often accompanied by a ‘hypocorticortisolemic symptom triad’ of heightened vigilance, fatigue and pain [3]. These chronic stress conditions are often associated with reduced—rather than enlarged—amygdala volumes [57–59], putatively due to neurotoxic damage following excessive cortisol exposure [60]. As the ‘non-responder’ trajectory comprised a large group of mixed participants, which did not report more childhood trauma, life events or psychiatric symptoms than other groups, our results unlikely represent such chronic forms HPA-axis dysregulation. An alternative would be that ‘non-responders’ were healthy, with a relative preservation of neural integrity due to lesser cortisol exposure [60]. Healthy adolescents—compared to depressed ones—also exhibited larger amygdala volumes and lower cortisol responses to stress [17].

This hypothesis is further supported by the fact that higher amygdala activation also increased the odds of these ‘non-responses’, though this effect became statistically non-significant after TBV-correction. As a central hub of the salience network, the amygdala is typically activated during stress, facilitating attentional reorientation toward potential threats [2, 25, 61], although recent reviews also note deactivations [25, 62]. So, while the amygdala was engaged by the stressor in this group, they did not release cortisol in response. This may index their ‘context sensitivity’ or the ability to correctly identify the presence and absence of stressor cues. Context sensitivity bottlenecks the selection of appropriate response strategies [63, 64], which may have led to the re-appraisal of the stressor as controllable, rendering coping resources released by cortisol unnecessary [65, 66].

Together, these results suggest that the ‘non-responder’ group may reflect a healthy group with intact neural integrity and appropriate stress-related activation of the amygdala. If so, the question remains whether larger amygdala volumes could represent hypertrophy in ‘hyper-responders’ but brain health in ‘non-responders’. Mean amygdala volumes across trajectories seem congruent with this possibility, as ‘non-responders’ displayed only lightly elevated volumes, while ‘hyper-responders’ exhibit pronounced elevations, compared to the ‘responder’ reference. So, while our results currently cannot delineate healthy ‘non-responses’ from unhealthy ones, they may provide an interesting foundation for future longitudinal designs.

### Hippocampus models

The best-fitting hippocampus model had three cortisol response trajectories which largely overlapped with those derived from the amygdala model. Higher hippocampus volume increased the odds of ‘hyper-responses’, though this association was no longer statistically significant when controlling for TBV. Furthermore, higher hippocampus activation increased the odds of an ‘elevated baseline’ trajectory, an effect which remained robust to TBV adjustment.

#### Hippocampus volume and ‘hyper-responses’

That larger hippocampus volumes predicted cortisol ‘hyper-responses’ aligns with its established roles in neurogenesis and GR-mediated feedback [3, 67]. The hippocampus is a computational hub detecting contextual cues and supervising the expression of stress responses via its hypothalamic efferents [14, 67]. It is biphasically affected by stress: while moderate levels increase excitability, neurogenesis, and memory function, stress chronicity leads to a reduction of dendrites, synapses, and neurogenesis in rodents [54]. Similarly, stress initially decreases GR feedback sensitivity, prolonging cortisol release, before chronic stress causes feedback hypersensitivity and global cortisol downregulation [3]. Considering our healthy sample, one interpretation is that this group exhibits enhanced neuroplasticity and diminished GR feedback sensitivity characteristic of early disease stages. This aligns with findings linking larger hippocampal volumes to subclinical anxiety [68], whose etiology involves enhanced contextual fear-learning [69]. However, as these are largely the same individuals as amygdala-model ‘hyper-responders’, the same difficulty disambiguating (un)healthy responses ensues. The alternative remains that this group comprises healthy males with normatively larger hippocampi and cortisol responses [39, 53]. The fact that the effect weakened after controlling for TBV would support this line of reasoning.

#### Hippocampus activation and the ‘elevated baseline’ trajectory

Finally, higher hippocampal activation increased the odds of exhibiting an ‘elevated baseline’ trajectory. Unlike the amygdala, the hippocampus is typically deactivated during stress [2, 61]. However, fMRI deactivation does not imply the absence of neuronal processing. Animal studies show that the hippocampus binds elements of stressful experiences through sparse and conjunctive encoding. When successful, this enhances contextual discrimination and reduces anxiety-like behaviors by narrowing the range of eliciting events [67]. Though fMRI cannot detect the presence of such sparse cellular activity, a tentative synthesis of our results is that greater activation observed in this group may indicate its absence. In animals, this is linked to memory overgeneralization and enhanced fear responses [67]. Moreover, the ‘elevated baseline’ group reported more childhood adversities and showed no cortisol response to the stressor, consistent with models proposing that cortisol non-responsivity emerges secondarily to prolonged (early) stress exposure [3, 70]. In the long run, these factors may confer vulnerability to developing disorders marked by flattened cortisol and memory overgeneralization, such as PTSD [3, 71] or chronic pain [3, 72]. Indeed, trauma-exposed individuals with sustained threat-related hippocampal activation prospectively developed more PTSD symptoms [73]. These speculations, however, should be considered in the context of otherwise healthy individuals, which did not report greater subjective stress. Alternatively, this group may have exhibited a transient stress response upon entering the MRI, but subsequently disengaged, with hippocampal activation reflecting mind wandering or self-referential processes [74]. Again, in the absence of longitudinal follow-ups, our results cannot distinguish vulnerability markers from those unrelated to stress.

### Clinical relevance

Changes in limbic regions and cortisol stress responses have both been associated with the development of disease. Both have been approached using biphasic models, where initial increases in volume and cortisol responses are followed by a later reduction [3, 54, 68]. While much is known about the endpoints of the stress continuum—acute stress and stress-related disease—less is known about the transitioning phase, where regulatory systems begin to recalibrate [3]. Here we show initial links between limbic regions and cortisol response trajectories in healthy humans. We hypothesize that initial increases in limbic volume and feedback insensitivity may drive excess cortisol release observed in this study. Over time, this may result in compensatory mechanisms causing limbic atrophy and cortisol non-responsivity, which we did not observe in this healthy cohort. Though our findings currently cannot distinguish brain health from hypertrophy, future cohorts may investigate these potentially dynamic changes in samples entering a stressful phase, such as when transitioning to university, by repeatedly probing limbic volume and laboratory stress responses.

### Exploratory affect analyses

In our exploratory affect analyses, neither amygdala- nor hippocampus-derived cortisol trajectories differed in stress-induced negative affect. Similarly, ROI-derived negative affect trajectories did not overlap with cortisol-based response trajectories. These findings align with a body of literature reporting no or inconsistent associations between cortisol and affective stress responses [9, 11, 12]. In fact, the two systems may even be functionally dissociated, as dexamethasone administration prior to psychosocial stress induction suppressed cortisol reactivity but left affective responses intact [75, 76].

In ROI-derived negative affect models, amygdala, hippocampus, and rACC parameters did not predict trajectories, but mOFC parameters did. The mOFC model had four trajectories ranging from ‘slight-brief’ to ‘moderate-lasting’ negative affect, which paralleled longer term measures of stress and affect. Greater mOFC thickness increased the likelihood of ‘mild-lasting’ negative affect, aligning with the mOFC’s role in emotion regulation via inhibitory projections to the amygdala [77]. In our sample, the ‘mild-lasting’ trajectory reported higher levels of childhood trauma, while their psychiatric symptoms remained in the mild-to-moderate range [46, 47]. This aligns with definitions of resilience as mental health despite adversity [78] and with stress inoculation theories positing that moderate levels of early adversity may foster later resilience [79]. Indeed, squirrel monkeys exposed to controlled stressors early in life exhibited fewer anxiety-like behaviors in adulthood, which was accompanied by greater mOFC-amygdala connectivity [80]. Human studies have also linked greater mOFC thickness to reduced trait anxiety following childhood adversity [81] and resilience in adolescents exposed to peer abuse [82]. Together, these results suggest that greater mOFC integrity fosters acute affect regulation, which in turn may support resilience over time.

In sum, our results further corroborate a dissociation between cortisol and affective responses to stress. While cortisol trajectories were best explained by adding limbic parameters, affective trajectories were predicted by mOFC structure. Particularly, higher mOFC thickness was linked to 'mild lasting' negative affect in a potentially resilient group. These findings align with previous work positioning the mOFC as a key site for affect regulation and resilience.

### Limitations and future direction

Despite the considerable strengths of our study, including a large sample size and data-driven LCMM-approach to investigate cortisol variability alongside limbic and mPFC (s/f)MRI parameters, several limitations should be acknowledged. First, though we included *sex* as a covariate, which accounted for systematic sex differences, hyper-response trajectories nevertheless consisted exclusively of males, which generally release more stress-related cortisol [39]. To circumvent such conflations, studies in larger samples may aim to investigate sexes separately and/or include hormonal status as a covariate [83].

Secondly, deviant cortisol trajectories (‘hyper-responses’, ‘non-responses‘, ‘elevated baseline’) included only a small number of participants in relation to the overall sample, which could indicate overfitting and limit generalizability [43]. However, since the distribution was similar to other samples [8, 84] and one would expect only few participants to exhibit aberrant cortisol responses in a vigorously screened healthy cohort, we believe this may also reflect the sensitivity of LCMM analyses, rather than a limitation per se. Future studies may address this issue by replicating trajectories in independent cohorts, or by investigating larger and more diverse samples.

Thirdly, though we had planned to investigate lateralization effects, convergence issues led us to merge ROIs across hemispheres. However, as lateralization effects were reported in both functional [85] and structural [86] investigations of acute stress, larger studies should include interaction terms for hemispheres instead.

Furthermore, exposure-time effects did not predict cortisol or affect trajectories. Changes over the course of stress exposure may thus not explain additional variance beyond mean activation in healthy samples. However, since such neural sensitization and habituation effects were shown in clinical samples [20, 23–25], exposure-time effects may prove relevant in future clinical investigations.

Additionally, the emergence of near-parallel lines in our mOFC negative affect model may suggest that a single trajectory was coerced into four levels, known as the ‘salsa effect’ (low, medium, hot) [43]. In this case, the identified trajectories may represent a baseline cognitive bias, rather than qualitative differences in acute stress appraisal. To date, few studies have investigated stress-related affect trajectories, e.g. [12], so the significance of our affect trajectories awaits replication.

Moreover, instead of estimating separate cortisol and affect models, investigations combining both outcomes found it was exactly the mismatch (flattened cortisol with heightened negative affect) which was informative of clinical status [87]. Exploiting integrational methods such as *multlcmm* [41] may therefore pose an exciting future avenue to advance the understanding of cortisol-affect dissociations.

Furthermore, we found a one-trajectory baseline solution, while previous studies reported multiple-trajectory solutions at baseline [8, 12, 13]. Reasons may include their use of in-person laboratory stressors and omission of one-trajectory models. Our findings may therefore be limited to the scanning environment and similar comparison conditions. To assess the generalizability of cortisol trajectories, future work may explicitly compare different paradigms and analytic approaches.

Finally, utilizing the standard questionnaire battery implemented with Scan*STRESS* may not have been most appropriate approach for characterizing trajectories. Especially the *CTQ* has been critiqued for its incompleteness regarding range and timing of exposure [88] and conflation with recall bias [89]. To better explore associations between childhood adversity and cortisol trajectories, future investigations should characterize adversity using more comprehensive instruments [90].

## Conclusion

The identification and prediction of stress response trajectories was improved by adding brain parameters from the amygdala, hippocampus, and mOFC. While limbic parameters informed cortisol responses, mOFC parameters informed both acute and long-term negative affect responses. Though these findings provide first insights into how MRI-derived parameters of HPA-regulating regions may relate to aberrant cortisol and affect responses, the functional meaning of these results awaits disambiguation in longitudinal designs.

## Supplementary information

Supplemental Material Lipka et al.

## Data Availability

The datasets generated and/or analyzed during the current study are available from the corresponding author on reasonable request. A repository of studies that have already used and published the data is available here: https://osf.io/echja/.

## Code availability

The R code of *lcmm* analyses is provided here: https://osf.io/gk6w9/.
