# Supplementary material for: Predicting stress response trajectories: Differential contributions of limbic and prefrontal regions to cortisol and affective responses
Source: Transl Psychiatry. 2026 Jun 12;16:310. doi: 10.1038/s41398-026-04140-0 (PMC13263346; doi:10.1038/s41398-026-04140-0)
Supplement: Supplementary file 1 — Supplemental Material Lipka et al. [file 41398_2026_4140_MOESM1_ESM.pdf]

Supplementary Material: Predicting stress response trajectories: Differential contributions of limbic and prefrontal regions to cortisol and affective responses

Renée Lipka, MSc[1,2,3,4], Ludwig Kreuzpointner, PhD[5], Christoph Bärtl, PhD[1,4], Marina Giglberger, PhD[5], Julian Konzok, PhD[5], Hannah L. Peter, PhD[5], Nina Speicher, PhD[5], Lea Waller, MSc[1,4], Brigitte M. Kudielka, PhD[5], Stefan Wüst, PhD[5], Henrik Walter, MD, PhD[1,4] & **Gina-Isabelle Henze, PhD**[1,4]

[1]Department of Psychiatry and Neurosciences CCM, Charité – Universitätsmedizin Berlin, Corporate Member of Freie Universität Berlin, Humboldt-Universität zu Berlin, and Berlin Institute of Health, Berlin, Germany
[2]Department of Psychiatry and Neuroscience, Charité – Universitätsmedizin Berlin, CBF, Berlin, Germany
[3]Berlin School of Mind and Brain, Humboldt Universität zu Berlin, Berlin, Germany
[4]German Center for Mental Health (DZPG), Partner Site Berlin - Potsdam, Berlin, Germany
[5]Institute of Psychology, University of Regensburg, Regensburg, Germany

Corresponding author: Gina-Isabelle Henze, gina-isabelle.henze@charite.de

Contents

Brain model supplements 3

Figure S1. Correlation matrix of left and right parameter estimates of each region of interest (ROI) 3

Table S2. Mean amygdala predictor values across amygdala-derived cortisol trajectories 4

Table S3. Characterization of amygdala model-derived cortisol trajectories 4

Table S5. Mean hippocampus predictor values across hippocampus-derived cortisol trajectories ... 5

Visual cortex regional specificity analyses 5

TBV subcortical sensitivity analyses 7

Exploratory affect analyses 9

Figure S4. Negative affect across cortisol trajectories 10

Figure S5. Baseline and amygdala negative affect models 11

Figure S6. Alluvial plots depicting participant flow between amygdala cortisol and negative affect models 11

References 13

Brain model supplements

Hormonal status

Our brain models were controlled for sex and not hormonal status (i.e., cycle phase or contraceptive use) even though prior work showed cortisol differences as a function of these variables (1). This is because repeated-measures analyses of variance (ANOVAs) in our sample indicated that while there were differences in cortisol between sexes (main effect of time: $F(3.08, 797.33) = 67.33, \eta_p^2 = .20$, main effect of sex: $F(1, 259) = 18.31, p < 0.01, \eta_p^2 = .06$, sex*time interaction: $F(3.08, 797.33) = 17.02, p < .001, \eta_p^2 = .06$), no differences were observed between cycle phases of females (main effect of time: $F(3.02, 431.77) = 16.79, p < .001, \eta_p^2 = .11$), main effect of cycle: $F(1, 143) = .15, p = .69, \eta_p^2 = .00$, cycle*time interaction: $F(3.02, 431.77) = 1.70, p = .16, \eta_p^2 = .01$).

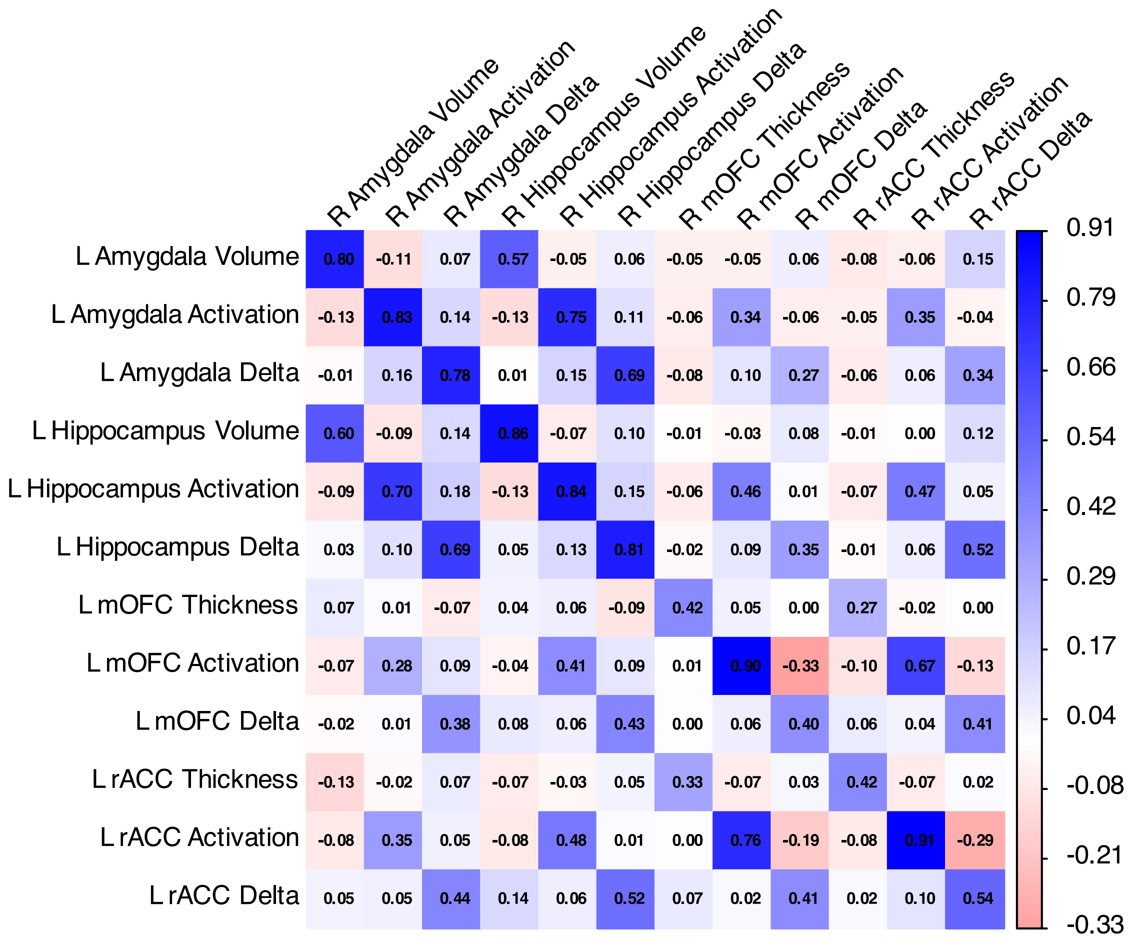

Figure S1. Correlation matrix of left and right parameter estimates of each region of interest (ROI). Note that the delta parameter refers to the ‘exposure-time effect’. Abbreviations: L, left; R, right; mOFC, medial orbitofrontal cortex; rACC, rostral anterior cingulate.

Table S1. Trajectory membership prediction of the 4-trajectory amygdala cortisol model

**Fixed effects per trajectory**	**Coef**	**SE**	**Wald**	***p***
Amygdala activation hyper-responder	0.86	0.71	1.21	0.22
Amygdala activation elevated baseline	25.20	22.08	1.14	0.25
Amygdala activation non-responder	0.68	0.34	1.97	0.04*
Amygdala delta hyper-responder	-0.14	0.46	-0.31	0.75
Amygdala delta elevated baseline	-1.24	2.27	-0.54	0.58
Amygdala delta non-responder	0.18	0.23	0.80	0.42
Amygdala volume hyper-responder	1.96	0.73	2.68	0.00*
Amygdala volume elevated baseline	13.78	12.10	1.13	0.25
Amygdala volume non-responder	0.93	0.37	2.50	0.01*

Notes: The reference trajectory was the 'responder' trajectory. Grey shading indicates statistically significant trajectory-membership predictors. Abbreviations: SE = standard error.

Table S2. Mean amygdala predictor values across amygdala-derived cortisol trajectories

	Trajectory mean (SD)						
	Hyper-responder	Elevated baseline	Non-responder	Responder	Statistic(df)	*p*	η^2
Volume (mm^3)							
L	1588.23 (179.31)	1507.38 (122.97)	1455.07 (157.41)	1332.69 (138.93)	F(1, 279) = 79.55	< .001*	.22
R	1734.60 (159.73)	1691.49 (177.87)	1595.58 (154.57)	1485.72 (150.18)	F(1, 279) = 71.24	< .001*	.20
Activation (z)							
L	-.03 (.03)	.04 (.03)	-.01 (.03)	-.03 (.04)	F(1, 279) = 10.89	< .001*	.04
R	-.02 (.03)	.05 (.03)	-.01 (.03)	-.02 (.03)	F(1, 279) = 17.02	< .001*	.06
Delta (z)							
L	-.06 (.04)	-.03 (.05)	-.04 (.06)	-.05 (.06)	F(1, 279) = .47	.49	.00
R	-.05 (.04)	-.04 (.06)	-.03 (.06)	-.05 (.06)	F(1, 279) = 2.88	.09	.01

Abbreviations: L, left; R, right. *Please see Figures 2C-E for pairwise comparisons. Grey shading indicates significant differences between trajectory means.

Table S3. Characterization of amygdala model-derived cortisol trajectories

	Trajectory mean (SD)						
	Hyper-responder	Elevated baseline	Non-responder	Responder	Statistic(df)	p	Effect size
Age	23.56 (± 3.68)	27.42 (± 6.76)	25.31 (± 6.82)	25.20 (± 8.25)	F(3, 277) = 0.61	.60	η^2 = .01
Sex					$\chi^2(3)$ = 33.31	< .001	V = .34
M	18	10	36	72			
F	0	2	29	114			
BDI-II	11.78 (±12.60)	12.58 (±10.57)	11.17 (±9.71)	14.25 (±12.07)	F(3, 271) = 1.27	.28	η^2 = .01
TICS	15.39 (±9.62)	15.92 (±10.12)	16.37 (±8.85)	18.53 (± 9.60)	F(3, 275) = 1.41	.24	η^2 = .02
ASI (Total)	14.59 (±6.73)	17.00 (±9.30)	18.94 (±11.21)	20.95 (±10.98)	F(3, 272) = 2.41	.06	η^2 = .03
CTQ (Total)	31.06 (±7.49)	35.33 (±10.66)	31.48 (±5.04)	32.41 (±7.95)	F(3, 273) = 1.09	.35	η^2 = .01
LEC (Total)	69.94 (±6.38)	68.00 (±9.54)	66.88 (±10.66)	67.08 (±13.53)	F(3, 277) = 0.33	.80	η^2 = .00

Abbreviations: ASI = Anxiety Sensitivity Index, BDI-II = Beck Depression Inventory II, CTQ = Childhood Trauma Questionnaire, LEC = Life Events Checklist, TICS = Trier Inventory for the Assessment of Chronic Stress (Screening Scale). Grey shading indicates significant differences between trajectory means.

Table S4. Trajectory membership prediction of the 3-trajectory hippocampus cortisol model

Fixed effects per trajectory	Coef	SE	Wald	*p*
Hippocampus activation hyper-responder	0.00	0.33	0.02	0.97
Hippocampus activation elevated baseline	2.57	1.02	2.51	0.01*
Hippocampus delta hyper-responder	-0.18	0.40	-0.46	0.64
Hippocampus delta elevated baseline	0.77	0.74	1.04	0.29
Hippocampus volume hyper-responder	0.80	0.37	2.14	0.03*
Hippocampus volume elevated baseline	1.40	0.76	1.82	0.06

Notes: The reference trajectory was the 'mild-responder' trajectory. Grey shading indicates statistically significant trajectory-membership predictors. Abbreviations: SE = standard error.

Table S5. Mean hippocampus predictor values across hippocampus-derived cortisol trajectories

	Trajectory mean (SD)					
	Hyper-responder	Elevated baseline	Mild-responder	Statistic(df)	*p*	*η²*
Volume (mm³)						
L	4256.93 (350.33)	4241.36 (252.12)	3943.44 (366.57)	F(1, 279) = 13.58	< .001*	.05
R	4262.74 (285.68)	4313.63 (225.90)	4028.26 (375.54)	F(1, 279) = 8.76	< .001*	.03
Activation (z)						
L	-.03 (.03)	.02 (.02)	-.02 (.03)	F(1, 278) = 1.02	.31	.00
R	-.02 (.02)	.03 (.02)	-.02 (.03)	F(1, 278) = 4.56	.03*	.02
Delta (z)						
L	-.06 (.03)	.00 (.05)	-.04 (.05)	F(1, 278) = .06	.80	.00
R	-.04 (.03)	-.00 (.04)	-.04 (.05)	F(1, 277) = .55	.46	.00

Abbreviations: L, left; R, right. *Please see Figures 3C-E for pairwise comparisons. Grey shading indicates significant differences between trajectory means.

Visual cortex regional specificity analyses

To test the regional specificity of our cortisol findings, we ran two additional sets of models, using data from the visual cortex as predictors of trajectory membership, namely, the pericalcarine cortex (or primary visual cortex) and cuneal cortex. Models were again age- and sex-corrected. We had to remove exposure-time effect predictors in these models as model convergence failed due to numerical instability. This was likely caused by near-zero activation change across runs for these regions leading to non-identifiable parameters. Therefore, the visual cortex models included only cortical thickness and mean activation across runs as predictors of trajectory membership. Models are available here: https://osf.io/gk6w9/.

Results showed that none of the visual cortex models improved model fit over the baseline model without brain predictors (Table S6). When nevertheless exploring the four-trajectory solutions of these models, they yielded comparable results across visual cortical regions (except for some label switching, Figure S2). Some of the identified trajectories were visually comparable to the ones found using amygdala and hippocampus predictors, such as 'responder' and 'hyper-responder' profiles. However, other trajectories were different, exhibiting two 'non-response' profiles, rather than one 'non-response' and one 'elevated baseline' trajectory identified in amygdala and hippocampus models. Additionally, visual cortex parameters did not predict trajectory membership like amygdala and hippocampus parameters did (Table S7).

Together, these findings suggest that (1) adding visual cortex parameters did not improve model fit in the way amygdala and hippocampus parameters did, and (2) while within these worse-fitting models some cortisol trajectories were visually comparable to amygdala and hippocampus-identified trajectories, visual cortex parameters did not predict these trajectories. This suggests that the association between brain parameters and cortisol trajectories is specific to the selected stress-relevant ROIs in the amygdala and hippocampus, rather than reflecting a brain-wide effect.

Table S6. Between model comparison of visual cortex-informed cortisol models

Model	Loglik[1]	Npm[2]	AIC	BIC	SABIC	Entropy	Trajectory size (%) 1	2	3	4
**Baseline model**										
1-Trajectory	-4629.33	14	**9286.67**	**9337.61**	**9293.22**	**1.00**	100	NA	NA	NA
**Pericalcarine cortex models**										
2-Trajectory	-4628.29	18	9292.58	9358.07	9300.99	.83	3.91	96.08	NA	NA
3-Trajectory	-4623.84	23	9293.68	9377.37	9304.43	.63	12.81	80.42	6.76	NA
4-Trajectory	-4618.13	28	9292.26	9394.13	9305.35	.72	7.11	45.11	39.14	7.82
**Cuneal cortex models**										
2-Trajectory	-4628.30	18	9292.60	9358.09	9301.01	.97	97.15	2.84	NA	NA
3-Trajectory	-4624.62	23	9295.25	9378.93	9306.00	.91	91.45	2.84	5.69	NA
4-Trajectory	-4619.32	28	9294.65	9396.53	9307.74	.40	44.48	44.48	7.47	7.82

[1]Log likelihood, [2]Number of parameters, bold faced parameters indicate best fit among all displayed models. Grey shading indicated the overall best performing model.

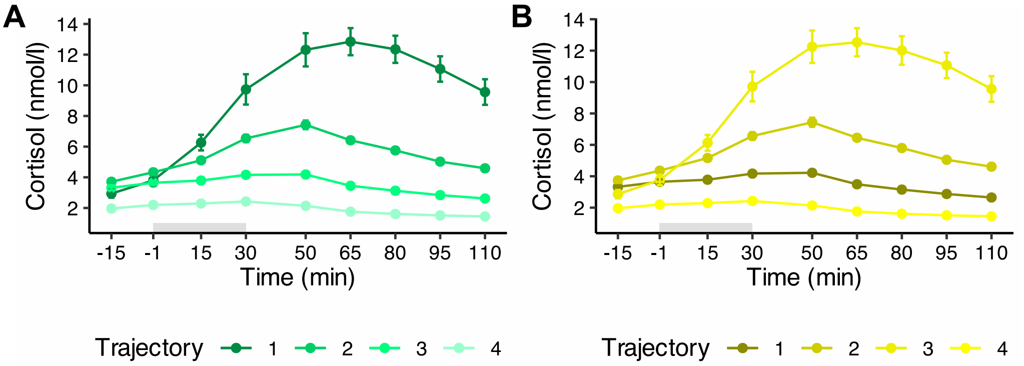

Figure S2. Cortisol trajectories of the four-trajectory visual cortex models. A) pericalcarine cortex-informed cortisol trajectories B) cuneal cortex-informed cortisol trajectories. We do note that these models had a worse fit than the single-trajectory baseline model without additional brain predictors and merely display them as proof of concept. Neither pericalcarine- nor cuneal cortex parameters (activation and thickness) were predictive of cortisol trajectory membership.

Table S7. Trajectory membership prediction of visual cortex models

**4-Trajectory pericalcarine cortex model**				
**Fixed effects per trajectory**	**Coef**	**SE**	**Wald**	***p***
Pericalcarine activation 1	-6.14	10.24	-0.60	0.54
Pericalcarine activation 2	1.13	7.28	0.15	0.87
Pericalcarine activation 3	10.02	8.20	1.22	0.22
Pericalcarine thickness 1	0.63	0.46	-1.35	0.17
Pericalcarine thickness 2	0.23	0.32	0.71	0.47
Pericalcarine thickness 3	0.07	0.37	0.20	0.83
**4-Trajectory cuneal cortex model**				
**Fixed effects per trajectory**	**Coef**	**SE**	**Wald**	***p***
Cuneus activation 1	12.57	8.04	1.56	0.11
Cuneus activation 2	3.53	7.46	0.47	0.63
Cuneus activation 3	0.15	9.69	0.01	0.98
Cuneus thickness 1	-0.05	0.30	-0.19	0.84
Cuneus thickness 2	0.06	0.28	0.22	0.81
Cuneus thickness 3	-0.57	0.38	-1.47	0.14

Notes: The reference trajectory is the fourth trajectory. Abbreviations: SE = standard error.

TBV subcortical sensitivity analyses

To verify that cortisol findings from the amygdala and hippocampus were not driven by total brain volume (TBV), we ran the two sets of models again, this time using TBV as an additional trajectory membership predictor. Models remained the same otherwise: including *sex* and *age* as covariates and regional activation, exposure-time effects, and volumes as predictors of trajectory membership. All predictors were z-standardized prior to model estimation to account for differences in scale. TBV models had convergence difficulties and multiple random starting vectors had to be attempted even after *gridsearch*. The converged models were highly similar to models without TBV as a trajectory membership predictor. Fit indices favored the same trajectory numbers: AIC and SABIC favored the four-trajectory amygdala model and AIC favored the three-trajectory hippocampus model (Table S8). Visual inspection of the resulting trajectories also closely matched those of models without TBV as a trajectory predictor (Figure S3). Amygdala models with and without TBV had near-identical trajectory assignments, only one participant switched from the 'hyper-responder' to the 'responder' trajectory: $Cohen's\ kappa = 0.97, z = 22.0, p < .001$. Hippocampus models with and without TBV also had near-identical trajectory assignment: $Cohen's\ kappa = 0.97, z = 21.0, p < .001$, with one participant switching from the 'elevated baseline' to the 'responder' trajectory. Trajectory membership prediction via multinomial logistic regression indicated increased uncertainty of some trajectory-membership estimates, reflected in inflated coefficient estimates and wider confidence intervals. The effects of amygdala and hippocampus parameters remained unchanged in their direction, though two were no longer statistically significant (Table S9). TBV-corrected models are available at: https://osf.io/gk6w9/.

Together, this suggests that our subcortical results were largely robust to the adjustment of TBV. Fit indices favored the same trajectory numbers, shapes and assignments remained highly similar, and trajectory predictor effects remained directionality consistent (though less precise and some no longer reached statistical significance).

Table S8. Between model comparison of subcortical cortisol models with total brain volume as an additional trajectory membership predictor

Model	Loglik[1]	Npm[2]	AIC	BIC	SABIC	Entropy	Trajectory size (%)			
							1	2	3	4
**Baseline model**										
1-Trajectory	-4629.33	14	9286.67	**9337.61**	9293.22	**1.00**	100	NA	NA	NA
**Amygdala models**										
2-Trajectory	-4624.46	20	9288.93	9361.70	9298.28	.86	95.01	4.89	NA	NA
3-Trajectory	-4617.84	27	9289.68	9387.91	9302.30	.57	6.76	60.85	32.38	NA
4-Trajectory	-4603.88	34	**9275.77**	9399.47	**9291.66**	.71	6.04	4.27	23.13	66.54
**Hippocampus models**										
2-Trajectory	-4626.20	20	9292.41	9365.18	9301.76	.88	3.55	96.44	NA	NA
3-Trajectory	-4616.20	27	**9286.40**	9384.64	9299.02	.86	3.91	3.91	92.17	NA
4-Trajectory	-4611.87	34	9291.74	9415.44	9307.63	.66	3.55	60.85	6.04	29.53

[1]Log likelihood, [2]Number of parameters, bold faced parameters indicate best fit relative to the baseline model. Grey shading indicates the selected amygdala and hippocampus models.

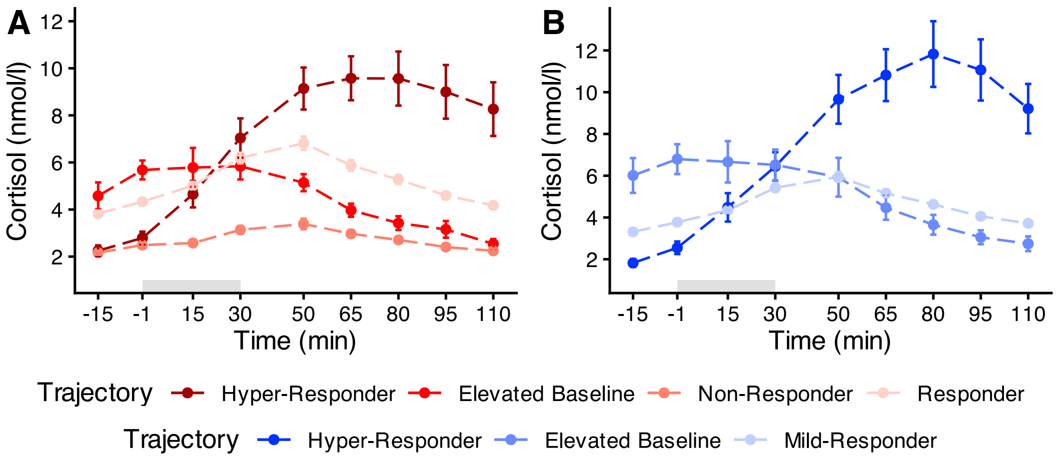

Figure S3. Mean cortisol of amygdala (A) and hippocampus (B) models with total brain volume as an additional trajectory membership predictor.

Table S9. Trajectory membership prediction of subcortical cortisol models with total brain volume as an additional trajectory membership predictor

**4-trajectory amygdala model**				
**Fixed effects per trajectory**	**Coef**	**SE**	**Wald**	***p***
Amygdala activation hyper-responder	17.21	23.42	0.73	0.46
Amygdala activation elevated baseline	574.37	464.95	1.23	0.21
Amygdala activation non-responder	18.41	10.04	1.83	0.06
Amygdala delta hyper-responder	-0.63	9.26	-0.06	0.94
Amygdala delta elevated baseline	-19.83	36.45	-0.54	0.58
Amygdala delta non-responder	3.40	4.09	0.83	0.40
Amygdala volume hyper-responder	2.39	1.04	2.28	0.02*
Amygdala volume elevated baseline	11.51	9.54	1.20	0.22
Amygdala volume non-responder	1.06	0.51	2.05	0.04*
TBV hyper-responder	-0.48	0.77	-0.61	0.53
TBV elevated baseline	-0.24	1.57	-0.15	0.87
TBV non-responder	-0.13	0.46	-0.29	0.76
**3-trajectory hippocampus model**				
**Fixed effects per trajectory**	**Coef**	**SE**	**Wald**	***p***
Hippocampus activation hyper-responder	-2.52	15.61	-0.16	0.87
Hippocampus activation elevated baseline	85.45	35.27	2.42	0.01*
Hippocampus delta hyper-responder	-3.53	7.98	-0.44	0.65
Hippocampus delta elevated baseline	15.63	14.91	1.04	0.29
Hippocampus volume hyper-responder	0.61	0.47	1.31	0.19
Hippocampus volume elevated baseline	1.51	0.86	1.74	0.08
TBV hyper-responder	0.25	0.45	0.56	0.57
TBV elevated baseline	-0.21	0.72	-0.30	0.76

Notes: The reference trajectory is the ‘responder’ trajectory for the amygdala model and the ‘mild-responder’ trajectory for the hippocampus model. Grey shading indicates trajectory-membership predictors that reached statistical significance in subcortical models without TBV as a trajectory predictor (refer to Tables S1 and S4 for direct reference). Abbreviations: SE = standard error, TBV = total brain volume.

Exploratory affect analyses

First, we explored whether cortisol trajectories from amygdala and hippocampus models differed in negative affect. A repeated-measures ANOVA on PANAS negative affect using cortisol groupings from the four-trajectory amygdala model revealed no significant differences between trajectories (main effect of time: $F(3.79, 963.27) = 45.44,\ p < .001, \eta_g^2 = .15$, main effect trajectory: $F(3.00, 254.00) = 1.31,\ p = .27, \eta_g^2 = .02$, time*trajectory interaction: $F(11.38, 963.27) = .82,\ p = .62, \eta_g^2 = .08$). See Figure S4A for a visualization. A repeated-measures ANOVA on negative affect with cortisol-derived trajectories from the hippocampus model also gave no indication for differential affect across cortisol trajectories (main effect time: $F(3.77, 961.95) = 18.66, p < .001, \eta_g^2 = .07$, main effect trajectory: $F(2.00, 255.00) = .35,\ p = .71, \eta_g^2 = .00$, time*trajectory interaction: $F(7.55, 961.95) = 1.12,\ p = .35,\ \eta_g^2 = .01$, Figure S4B).

When cortisol trajectories did not show utility in explaining variance in negative affect, we further explored whether re-running baseline and brain models with negative affect as the outcome may facilitate the identification of distinguishable affect trajectories (Table S10). Among baseline models, all fit indices unanimously (BIC, SABIC, AIC) selected the three-trajectory model with low entropy ($.48$). The first trajectory exhibited 'moderate-lasting' negative affect in response to stress ($N = 78$), the second trajectory had a mild-brief negative affective response ($N = 100$), and the final trajectory had a slight-brief negative response ($N = 101$). The baseline affect model is displayed in Figure S5A.

In the following, we compared this winning baseline model to brain models. For amygdala, hippocampus, and rostral anterior cingulate cortex (rACC) models, BIC, SABIC, and entropy indices ($all\ .81$) favored two-trajectory models, while AIC favored the three-trajectory models. Hence, we explored both. The two-trajectory models of all three ROIs converged on similar points (Table S10), leading to identical trajectory assignment across models ($Fleiss'\ kappa = 1,\ z = 28.9,\ p < .01$). Descriptively, the first trajectory showed a 'moderate negative affect' trajectory ($N = 71$) and the second a 'mild negative affect' trajectory ($N = 208$). The Polynomial logistic regression model indicated that brain parameters of the three ROIs showed no utility in predicting membership of two-trajectory affect models ($all\ p's > .05$). The same was true for four-trajectory models. Convergence points of the three ROIs were similar (Table S10) and so were trajectory assignments ($Fleiss'\ kappa = .89,\ z = 42.2,\ p < .001$). Descriptively, the four trajectories followed similar patterns, but varied in intensity, from 'moderate-lasting' negative affect in the first trajectory towards 'slight-brief' negative affect in the fourth trajectory. The first trajectory comprised the smallest sample portion ($N = 28$), while trajectories two to four had approximately equal proportions, with $N = 80, 83, 88$, respectively. Additionally, none of the ROIs predicted the four negative affect trajectories in the polynomial logistic regression part of the analyses ($all\ p's > .05$). We therefore only display amygdala two- and four-trajectory models (Figure S5B&C). However, due to the similarity between models, amygdala affect models may also be considered as stand-ins for hippocampus and rACC models.

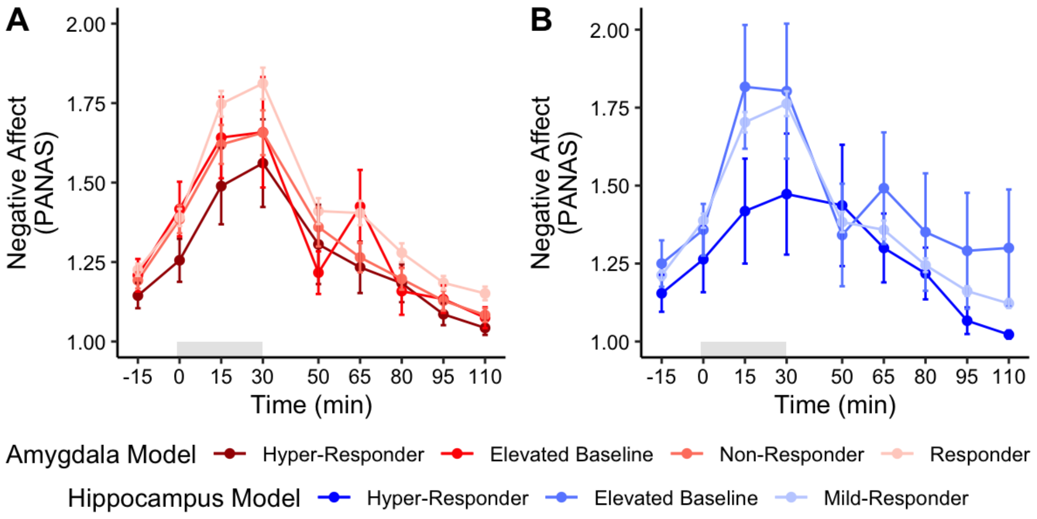

Figure S4. Negative affect across cortisol trajectories (A) from the 4-trajectory amygdala model and (B) the 3-trajectory hippocampus model. Abbreviations: PANAS = Positive and Negative Affect Schedule. Grey shading indicates the timing of Scan*STRESS*.

Table S10. Between model comparison of negative affect models

Model	Loglik[1]	Npm[2]	AIC	BIC	SABIC	Entropy	Trajectory size (%)			
							1	2	3	4
**Baseline models**										
1-Trajectory	-86.76	13	199.53	246.73	205.51	**1.00**	100	NA	NA	NA
2-Trajectory	-83.92	16	199.85	199.85	257.95	.44	63.08	36.91	NA	NA
3-Trajectory	-54.02	19	**146.05**	**215.04**	**215.04**	.48	27.95	35.84	36.20	NA
4-Trajectory	-54.02	22	152.04	231.93	231.93	.41	28.31	48.74	.00	22.93
**Amygdala models**										
2-Trajectory	-53.37	19	144.75	**213.74**	**153.49**	**.81**	25.44	74.55	NA	NA
3-Trajectory	-52.06	25	154.13	244.91	165.63	.51	45.16	27.24	27.59	NA
4-Trajectory	-39.39	31	**140.79**	253.35	155.06	.67	10.03	28.67	29.74	31.54
**Hippocampus models**										
2-Trajectory	-53.29	19	144.58	**213.57**	**153.33**	**.81**	25.44	74.55	NA	NA
3-Trajectory	-51.71	25	153.42	244.20	164.93	.51	27.24	21.50	51.25	NA
4-Trajectory	-38.87	31	139.75	252.32	154.02	.64	8.60	29.39	29.03	32.97
**mOFC models**										
2-Trajectory	-54.07	19	146.14	215.14	154.89	**.81**	25.44	74.55	NA	NA
3-Trajectory	-51.75	25	153.51	244.29	165.02	.54	27.24	45.51	27.24	NA
4-Trajectory	-34.40	31	**130.81**	243.38	**145.08**	.67	11.11	25.08	24.73	39.06
**rACC models**										
2-Trajectory	-53.12	19	144.25	**213.25**	**153.00**	**.81**	25.44	74.55	NA	NA
3-Trajectory	-48.96	25	147.92	238.70	159.43	.62	31.89	41.21	26.88	NA
4-Trajectory	-38.41	31	**138.83**	251.40	153.10	.66	11.46	25.80	31.89	30.82

[1]Log likelihood, [2]Number of parameters, grey shading indicates selected models.

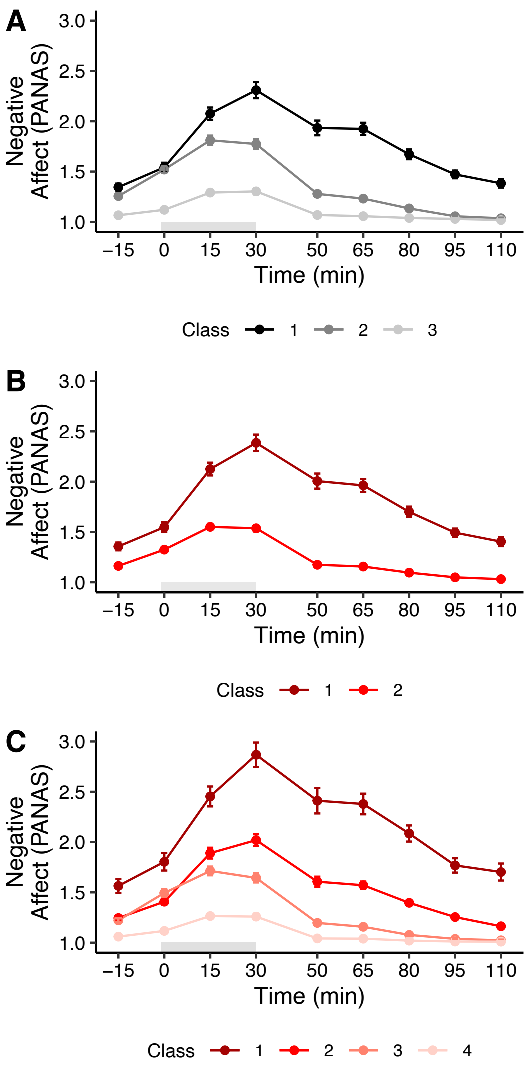

Figure S5. Baseline and amygdala negative affect models. A) Best fitting baseline model without additional brain predictors. B + C) Amygdala-derived two- and four-trajectory negative affect models. Note that amygdala plots may be read as surrogates for rACC and hippocampus affect models, which had nearly identical trajectories and assignments. Grey shading indicates Scan*STRESS* timing.

Furthermore, we explored whether cortisol trajectories from the selected amygdala and hippocampus models corresponded with negative affect models with the same trajectory numbers, as well as the winning two-trajectory affect model. There was no meaningful correspondence between cortisol and affect trajectory assignment for neither amygdala nor hippocampus models. See Figure S6 for an example of such non-correspondence in amygdala models.

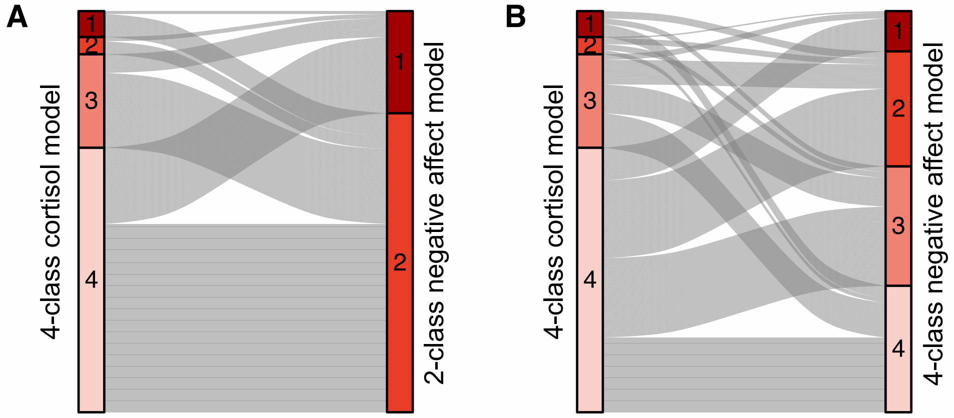

Figure S6. Alluvial plots depicting participant flow between amygdala cortisol and negative affect models. A) Best-fitting four-trajectory cortisol model and two-trajectory negative affect model. B) Best-fitting four-trajectory cortisol model and four-trajectory negative affect model.

Table S11. Trajectory membership prediction of the 4-trajectory mOFC negative affect model

**Fixed effects per trajectory**	**Coef**	**SE**	**Wald**	***p***
mOFC activation moderate-lasting	-0.34	0.25	-1.35	0.17
mOFC activation mild-lasting	0.20	0.20	0.98	0.32
mOFC activation mild-brief	-0.01	0.25	-0.05	0.95
mOFC delta moderate-lasting	-0.30	0.28	-1.06	0.28
mOFC delta mild-lasting	0.20	0.25	0.79	0.42
mOFC delta mild-brief	-0.29	0.32	-0.89	0.37
mOFC thickness moderate-lasting	-0.38	0.33	-1.14	0.25
mOFC thickness mild-lasting	0.64	0.29	2.17	0.02*
mOFC thickness mild-brief	0.58	0.34	1.70	0.08

Notes: The reference trajectory was the ‘slight-brief’ trajectory. Grey shading indicates statistically significant trajectory-membership predictors. Abbreviations: mOFC = medial orbitofrontal cortex, SE = standard error

References

1. Zänkert S, Bellingrath S, Wüst S, Kudielka BM (2019): HPA axis responses to psychological challenge linking stress and disease: What do we know on sources of intra- and interindividual variability? *Psychoneuroendocrinology* 105: 86–97.
